# Magnetic thermoradiotherapy for lung cancer: evaluation in A549−based preclinical models

**DOI:** 10.3389/fonc.2026.1828140

**Published:** 2026-05-11

**Authors:** Agnieszka Stawarska, Magdalena Bamburowicz-Klimkowska, Artur Kasprzak, Monika Ruzycka-Ayoush, Michal Bystrzejewski, Maria Wojewodzka, Michal Wieteska, Ireneusz P. Grudzinski

**Affiliations:** 1 Medical University of Warsaw, Faculty of Pharmacy, Department of Toxicology and Food Science, Warsaw, Poland; 2 Warsaw University of Technology, Faculty of Chemistry, Department of Organic Chemistry, Warsaw, Poland; 3 University of Warsaw, Faculty of Chemistry, Department of Physical Chemistry, Warsaw, Poland; 4 Institute of Nuclear Chemistry and Technology, Centre for Radiobiology and Biological Dosimetry, Warsaw, Poland; 5 Warsaw University of Technology, Institute of Radioelectronics and Multimedia Technology, Warsaw, Poland; 6 Mossakowski Medical Research Institute, Polish Academy of Science, Warsaw, Poland

**Keywords:** combination therapy, glucose oxidase, lung cancer, magnetic fluid hyperthermia, magnetic nanoparticles, monoclonal antibody, radiotherapy, αvβ3 integrin

## Abstract

**Background/Objectives:**

Radiotherapy is a cornerstone in lung cancer management; however, achieving effective tumor eradication while sparing surrounding healthy tissue remains a major clinical challenge. Recent advances in nanotechnology offer new opportunities to enhance radiotherapeutic precision and efficacy through improved tumor targeting, increased dose deposition, and integration of complementary treatment modalities such as magnetic fluid hyperthermia (MFH). This study evaluates a novel strategy combining X−ray irradiation, MFH, and engineered magnetic nanoparticles to improve treatment efficacy in lung cancer.

**Methods:**

Carbon-encapsulated iron nanoparticles (Fe@C), coated with polyethyleneimine (PEI) and functionalized with a monoclonal IgG antibody targeting the β3 subunit (CD61) of the αvβ3 integrin receptor, were loaded with glucose oxidase (GOX) to produce Fe@C-PEI-IgG-GOX nanoparticles. These nanoparticles were used to sensitize lung cancer cells prior to radiotherapy in combination with magnetic fluid hyperthermia induced by iron oxide nanoparticles exposed to an alternating magnetic field. Therapeutic efficacy was evaluated using human adenocarcinomic alveolar basal epithelial cells (A549) in both *in vitro* and *in vivo* models, including A549 cell cultures and NUDE Balb/c mice bearing A549 xenograft tumors.

**Results:**

The engineered Fe@C−PEI−IgG−GOX nanoparticles significantly reduced lung cancer cell viability *in vitro*, consistent with the known enzymatic activity of glucose oxidase, which catalyzes the oxidation of glucose to gluconic acid with concomitant hydrogen peroxide generation. This process negatively affected cancer cell survival, induced DNA damage, and suppressed colony formation. *In vivo*, the combination of radiotherapy and magnetic fluid hyperthermia applied together with a GOX-containing, anti-CD61-functionalized magnetic nanoplatform was associated with improved therapeutic outcomes in the lung cancer model. This multimodal approach resulted in enhanced therapeutic efficacy and prolonged time to the protocol−defined endpoint.

**Conclusions:**

Overall, these findings highlight the promise of a GOX-based, targeted magnetic nanoplatform combined with radiotherapy and magnetic fluid hyperthermia as an effective multimodal strategy for lung cancer treatment, warranting further preclinical optimization and mechanistic studies. Importantly, the observed therapeutic benefit reflects the overall outcome of the combined treatment in the *in vivo* model, and the relative contributions or potential interactions among the individual treatment modalities were not mechanistically dissected in this study.

## 1 Introduction

Lung cancer (carcinoma pulmonis) represents one of the most critical oncological entities in terms of both incidence and mortality. The etiopathogenesis of this tumor is multifactorial, with a predominant role of environmental factors, particularly exposure to tobacco smoke, which contains numerous carcinogens, including polycyclic aromatic hydrocarbons, nitrosamines, and heavy metal compounds. These substances induce DNA adduct formation, leading to mutations in tumor suppressor genes (TP53, RB1) as well as activation of proto-oncogenes (KRAS, EGFR) (1, 2). Environmental factors unrelated to smoking also play a significant role in lung carcinogenesis, such as exposure to radon, asbestos, arsenic, and fine particulate air pollution (PM2.5) (3, 4). Non-small cell lung cancer (NSCLC) accounts for approximately 85% of all lung cancer cases and represents a heterogeneous histogenetic group comprising three major subtypes: adenocarcinoma, squamous cell carcinoma, and large cell carcinoma (5). Adenocarcinoma, the most common NSCLC subtype, arises from the epithelial cells of the distal bronchioles and alveoli. Its development is frequently driven by the presence of driver mutations in EGFR, ALK, ROS1, BRAF, and KRAS, which activate the MAPK, PI3K/AKT, and JAK/STAT signalling pathways, ultimately leading to uncontrolled proliferation and resistance to apoptosis (5). Squamous cell carcinoma originates from the bronchial epithelium of the central airways and is characterized by aberrations in TP53, SOX2, and CDKN2A, which lead to dysregulation of cell-cycle control and differentiation. Large cell carcinoma, by contrast, is a highly anaplastic tumor lacking features of specific differentiation. A shared hallmark of all NSCLC subtypes is their strong capacity for invasion, angiogenesis, and metastasis, driven by the activation of epithelial-mesenchymal transition (EMT) pathways and the induction of pro-angiogenic factors such as VEGF. The molecular heterogeneity of NSCLC makes this malignancy one of the key subjects of contemporary translational oncology research (5, 6).

Radiotherapy constitutes one of the fundamental pillars in the treatment of lung cancer, serving both therapeutic and palliative purposes. In non-small cell lung cancer, for patients with early-stage disease who are not candidates for surgical resection, stereotactic body radiotherapy (SBRT/SABR) has become the standard of care. This technique enables the precise delivery of high radiation doses directly to the tumor while minimizing exposure to healthy lung tissue, resulting in high local control (LC) rates - ranging from 85% to 100% - and limited toxicity (7, 8). The literature emphasizes that SBRT currently represents a curative-intent therapy for patients who are not eligible for surgery, particularly in the case of peripheral tumors (7). In patients with locally advanced NSCLC, when surgical tumor removal is not feasible, concurrent chemoradiotherapy remains the standard of care. However, attempts to escalate the radiation dose to 74 Gy have not yielded a clear survival benefit in randomized trials such as RTOG 0617, highlighting the need for individualized treatment approaches that carefully consider both pulmonary and cardiac toxicity (7, 9). Technical aspects of radiotherapy, such as tumor respiratory motion and the proximity of critical structures, necessitate the use of advanced irradiation techniques, including 4D-CT imaging, conformal planning (IMRT, VMAT), tumor-motion tracking, and adaptive planning. These approaches enable precise dose delivery while minimizing damage to surrounding healthy tissues (7, 9). A major limitation of radiotherapy is tumor hypoxia, which reduces the effectiveness of ionizing radiation. Consequently, strategies aimed at modulating the tumor microenvironment – either by improving tumor oxygenation or by combining radiotherapy with biological therapies – are being actively developed. In advanced and oligometastatic NSCLC, radiotherapy is no longer confined to a purely palliative role; treatment models incorporating local irradiation in combination with systemic therapy have demonstrated improved local control and potentially enhanced overall survival (10).

In recent years, magnetic fluid hyperthermia has gained increasing importance as a strategy to enhance the effectiveness of lung cancer radiotherapy. This modern therapeutic technology employs the properties of ferromagnetic nanoparticles to generate heat within the tumor tissue (11). The mechanism of this method is based on the controlled heating of tumor tissues to a temperature of 41–45°C using magnetic nanoparticles, most commonly iron oxides, which, after being administered directly into the tumor (*in situ*) or intravenously, generate heat upon exposure to an external oscillating magnetic field with a frequency of 100–500 kHz. Elevation of the temperature within the 41–45°C range induces a series of biological effects, including destabilization of cell membranes, protein denaturation, disruption of mitochondrial function, and activation of caspases that initiate apoptosis (12–14). From a radiobiological perspective, magnetic hyperthermia functions as a potent radiosensitizer, enhancing the susceptibility of cancer cells to ionizing radiation. The synergistic effect between hyperthermia and radiotherapy arises primarily from the impairment of DNA damage–repair mechanisms, particularly the repair of double-strand breaks (DSBs), as well as from improved perfusion and oxygenation within the tumor microenvironment, which helps eliminate regions of radioresistant hypoxia (15, 16). Additionally, an increase in the expression of heat shock proteins (HSP70, HSP90) is observed, which modulate the immune response through mechanisms of immunogenic cell death (ICD). The combination of radiotherapy and magnetic hyperthermia, referred to as magnetic thermoradiotherapy, leads to an enhanced tumor-cell kill ratio without a proportional increase in toxicity to healthy tissues. Preclinical models have demonstrated that sequential application of hyperthermia 30–60 minutes after radiation exposure induces persistent inhibition of DNA repair pathways (ATM/ATR, BRCA1/2) and significantly reduces cell survival (15, 16). Moreover, the use of functionalized nanoparticles (e.g., those coated with ligands targeting EGFR receptors or αvβ3 integrins) enables precise delivery of the heating agent to cancer cells, while minimizing the risk of damage to healthy lung tissue (17). An increasing body of evidence in the literature supports the effectiveness of these approaches in preclinical lung cancer models. For example, one study demonstrated that the use of Mn–Zn ferrite nanoparticles (MZF-HA) in a subcutaneous tumor model derived from A549 lung adenocarcinoma cells resulted in a significantly reduced tumor volume when hyperthermia was combined with radiotherapy compared with the control group confirming the potential to enhance the efficacy of radiation therapy in the context of NSCLC (18). Furthermore, our own study demonstrated that, in an *in vitro* model using A549 cells and an *in vivo* xenograft model (A549) in mice, the application of magnesium-doped iron oxide nanoparticles (Mg_0.1_-γ-Fe_2_O_3_(mPEG silane)_0.5_) in combination with an external magnetic field resulted in a significant inhibition of tumor-volume growth at SAR values of 100–150 W·g^-1^ and a CEM43 of 9.6 min (19). Likewise, a study involving magnetic particles in the treatment of lung adenocarcinoma through the delivery of siRNA and antisense oligonucleotides (AS-ODN) demonstrated an improvement in radiotherapy efficacy attributable to the presence of magnetic nanoparticles (15). Therefore, in light of these findings, the combination of magnetic hyperthermia and radiotherapy is increasingly recognized as a promising combined-modality strategy for NSCLC, particularly in cases resistant to standard treatments. Moreover, the results of current studies suggest that the key parameters determining the efficacy of this approach include: adequate intratumoral distribution of nanoparticles (accumulation), effective heating capacity (SAR, CEM43), proper synchronization of the magnetic field with radiotherapy sessions, and the timing of hyperthermia relative to irradiation, for example, applying hyperthermia within 30–60 minutes after a selected radiation dose has been shown to induce significant inhibition of DNA repair pathways (ATM/ATR, BRCA1/2). The combination of radiotherapy and hyperthermia is an established radiosensitizing approach, whose mechanisms involve, among others, suppression of radiation-induced DNA damage repair and modulation of tumor hypoxia and perfusion, which may translate into improved local control without a proportional increase in toxicity when thermal parameters are appropriately managed (14, 16, 20, 21). From a translational perspective, new heat-delivery technologies such as RF/MW systems and nanomaterial-based approaches are being developed to enhance the selectivity and reproducibility of tumor heating, potentially broadening the applications of hyperthermia as an adjunct to radiotherapy (22, 23).

In the present study, we conducted an evaluation of the therapeutic efficacy of magnetic thermoradiotherapy in non-small cell lung cancer. The study employed carbon-encapsulated iron nanoparticles (Fe@C), coated with poly(ethylenimine) (PEI) and functionalized with a monoclonal IgG antibody specifically recognizing the β3 (CD61) subunit of the αvβ3 integrin receptor, which is overexpressed in lung cancer (24, 25). The nanoparticles were then loaded with glucose oxidase (GOX), yielding the Fe@C-PEI-IgG-GOX nanostructure. The as-loaded enzyme exhibits substantial potential for sensitizing cancer cells to radiotherapy, as it catalyses the oxidation of glucose with the generation of hydrogen peroxide (H_2_O_2_), thereby enhancing oxidative stress and increasing the levels of reactive oxygen species (ROS) (26). The increase in ROS promotes the intensification of radiation-induced DNA damage while simultaneously “starving” cancer cells through glucose consumption by GOX, which further diminishes their capacity to repair such damage (27, 28). The nanoparticles designed in this manner were used to sensitize non-small cell lung cancer cells to radiotherapy in combination with magnetic fluid hyperthermia triggered by iron oxide nanoparticles subjected to an alternating magnetic field. The effectiveness of the nanoparticles used as targeted GOX carriers and magnetic thermoradiotherapy was evaluated in both *in vitro* studies using the A549 cell line, as well as in a murine, immunodeficient lung cancer model, with implanted A549 tumors to NUDE Balb/c mice. Furthermore, magnetic resonance imaging (MRI) was used to assess radiological dosimetry and the spatial distribution of tumor burden in experimental animals, providing high−resolution, non−invasive insights into tumor volume and progression.

## 2 Materials and methods

### 2.1 Nanoparticles

**Figure 1:**
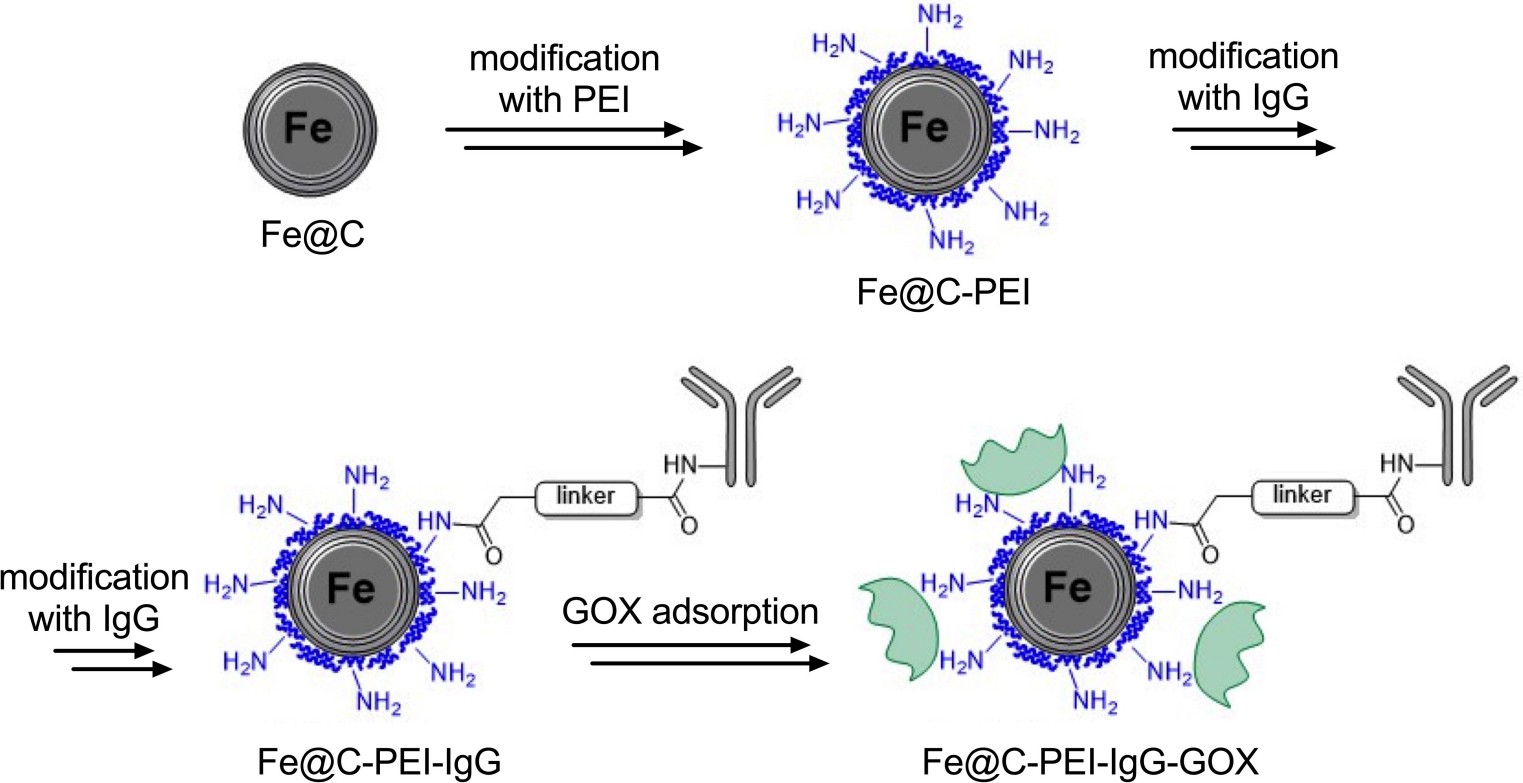
Carbon-encapsulated iron nanoparticles functionalized with polyethyleneimine (PEI) and anti-CD61 monoclonal IgG antibodies and loaded with glucose oxidase (GOX). Based on (31–33).

Carbon-encapsulated iron nanoparticles (Fe@C) were synthesized using the carbon arc discharge method as described earlier (29, 30). The synthesis was carried out under an Ar-H_2_ atmosphere (50:50 vol%) at 70 A and 60 kPa. The resulting particles were purified by refluxing in 3 M HCl, washing, and drying, yielding core–shell Fe@C nanoparticles sized between 10 and 100 nm. Surface modification of the as-obtained Fe@C nanoparticles with carboxylic groups allowed conjugation of polyethyleneimine through carbodiimide and radical-type reactions according to Kasprzak et al. (31). Subsequently, the PEI (Sigma-Aldrich, St. Louis, USA) attached to the Fe@C nanoparticles was derivatized to introduce sulfhydryl groups, enabling further bioconjugation studies. This sulfhydrylated Fe@C-PEI-SH nanoplatform was then used to conjugate human polyclonal IgG antibodies, through bioconjugation-type reactions resulting Fe@C-PEI-IgG nanoparticles (32). The same type of the conjugate reaction was used to attach mouse anti-human CD61 monoclonal IgG1 antibodies (Beckton Biosciences). Next, the immobilization of glucose oxidase (Serva GmbH, Germany) was achieved through the electrostatic attachment of the GOX enzyme onto Fe@C-PEI-IgG nanoparticles to yield Fe@C-PEI-IgG-GOX nanoparticles using the method recently described by Bamburowicz et al. (33). The synthesis scheme of these nanoparticles is shown in **Figure 1**. The nanoparticles were characterized as previously described for pristine (30), PEI-functionalized (31, 32, 34, 35), antibody-functionalized (31, 32), and GOX-loaded nanoparticles (33), respectively. Comprehensive magnetic resonance characteristics of Fe@C was described earlier by our group (30). Additionally, the ability of antibody-functionalized carbon encapsulated iron nanoparticles to target integrin receptors was recently described by Stawarska et al. (36).

### 2.2 Glucose oxidase

The activity of glucose oxidase was assessed in both its free and conjugated forms using a coupled assay method, following the procedure outlined by Cao et al. (37). In this assay, glucose was oxidized by GOX, resulting in the production of hydrogen peroxide (H_2_O_2_). The H_2_O_2_ agent then reacted with the chromogenic substrate 3,3′,5,5′-tetramethylbenzidine (TMB) in a reaction catalyzed by horseradish peroxidase (HRP). This led to the formation of a colored quinoneimine compound, whose absorbance was measured at a wavelength of 625 nm using 1-cm path length cuvettes and a Metertech UV–VIS SP 8001 spectrophotometer. Enzymatic activity was tracked over a 30-minute period following enzyme addition, and the initial reaction rate was determined from the linear portion of the resulting curve. All measurements were performed in triplicate, using free GOX as a reference. To determine the maximum reaction rate (*V*_max_) and the Michaelis–Menten constant (*K*_m_), the assay was conducted with varying glucose concentrations ranging from 5 to 50 µg/mL (equivalent to 27.8–277.5 nM).

### 2.3 Cell culture

Human adenocarcinomic alveolar basal epithelial cell line (A549) obtained from American Type Culture Collection (ATCC) were used in the study. The cells were routinely cultured at 37°C in a humidified atmosphere with 5% CO_2_ in flasks containing F-12 K medium (Kaighn’s modification of Ham’s F-12 medium (Gibco, Paisley, UK), supplemented with 10% fetal bovine serum (FBS; Gibco, Paisley, UK), streptomycin, 50 μg·mL^− 1^, amphotericin B, 1.25 μg·mL^− 1^, gentamicin, 50 μg·mL^− 1^, penicillin, 50 μg·mL^− 1^ (all from GIBCO, Paisley, UK). The medium was changed every third day. The cells were washed twice with phosphate-buffered saline (PBS) and incubated with 0.25% trypsin solution for 3 minutes at 37°C to detach the cells. Thereafter, the complete media were then added into the flask at room temperature to inhibit the effect of trypsin. The cells were washed twice by centrifugation and re-suspended in the complete fresh media for reseeding and growing in new cultures. The cells were counted using a hemocytometer and prepared in a sterile PBS solution to be subcutaneously injected into mice. In addition, an appropriate number of cells (according to the instructions) were plated for cytotoxicity assays.

### 2.4 Alamar blue assay

This assay was carried out according to the manufacturer’s instruction (Alamar Blue™ Cell Viability Reagent; Thermo Fisher Scientific, Life Technologies Corporation, Eugene, OR, USA). The A549 cells were trypsinized using a 0.25% trypsolution (Gibco, Paisley, UK) and plated in 96-well plates (Falcon; Corning, Durham, NC, USA) at a density of 10^4^ cells per well. After 24 h of cell adhesion, the A549 cells were exposed to increasing concentrations (0.1, 1, 5, 10, 25, 50, and 100 μg·mL^− 1^) of an aqueous solution of Fe@C-PEI-IgG or Fe@C-PEI-IgG-GOX nanoparticles and were further incubated for an additional 24 h or 48 h, while the control cells were incubated with the medium. The solution of the tested nanoparticles constituted 10% of the medium. After incubation, the control medium and the tested compounds were removed, and the cells were rinsed twice with PBS. Following this, 100 μL of Alamar Blue solution (10% [v/v] solution of Alamar Blue dye in fresh medium) was transferred to each well. After 3 h of incubation (37°C, 5% CO_2_, 90% humidity), the Alamar Blue fluorescence was quantified using an Epoch microplate reader (BioTek) at an excitation and emission wavelength of 560 and 590 nm, respectively. The viability of cells was expressed as fluorescence counts in the presence of the test compound, which was recalculated as a percentage of the control cells. Three replicates were performed for each group.

### 2.5 Colony forming efficacy assay

The A549 cells (200 cells per dish) were resuspended in 1 mL of fresh complete medium and seeded in 35-mm Petri dishes. After 24 h of incubation (37^°^C, 5% CO_2_, 90% humidity), the cells were treated with Fe@C-PEI-IgG (1, 5, 10 μg·mL^− 1^) or Fe@C-PEI-IgG-GOX (1, 5, 10 μg·mL^− 1^) nanoparticles. The concentration range of 1–10 µg·mL^-1^ was chosen to evaluate long−term antiproliferative effects rather than acute cytotoxicity. While short−term viability assays indicated acceptable cellular tolerance at lower concentrations, clonogenic analysis revealed complete inhibition of colony formation for Fe@C−PEI−IgG−GOX nanoparticles within this range, highlighting sustained suppression of proliferative capacity. This effect is consistent with GOX−mediated metabolic stress and impaired cellular recovery rather than immediate nonspecific toxicity. Positive control (sodium chromate, Na_2_CrO_4_, 100 μM), negative control (medium), and solvent control (medium containing 10% FBS) were used in parallel studies. The cells were treated for 72 h, and then, the incubated medium with nanoparticles was removed and completely replaced with a fresh culture medium. After the next 5 days the cells were fixed and stained using a fixing solution of 10% (v/v) formaldehyde in PBS and a staining solution of 10% (v/v) Giemsa in ultrapure water, respectively. The Petri dishes were air-dried before colony counting. The colonies were counted using a stereoscopic microscope (Opti-Tech Scientific). Three replicates were performed for each group.

### 2.6 Alkaline comet assay

The genotoxic potential of the tested nanoformulations was assessed using the comet assay (single-cell gel electrophoresis). A549 cells were seeded in 24-well plates at a density of 2.5 × 10^5^ cells per well and allowed to adhere for 24 h. Subsequently, the cultures were exposed to the IC_50_ concentrations of the Fe@C-PEI-IgG and Fe@C-PEI-IgG-GOX nanoconjugates and incubated for an additional 24 h. Untreated cells maintained in culture medium, or PBS served as negative controls. For the positive control, control cells were irradiated with 2 Gy X-rays in an ice-water bath using a Smart200 (Yxlon) system operating at 200 kV and 4.5 mA with 3-mm Al filtration (dose rate: 1.14 Gy/min). Following treatment, the cells were harvested by trypsinization, mixed 1:1 with 2% low-melting-point agarose (Type VII), and applied onto slides pre-coated with 0.5% standard agarose (Type I-A). Slides were transferred to lysis buffer, and DNA unwinding was performed in freshly prepared alkaline electrophoresis buffer (300 mM NaOH, 1 mM Na_2_EDTA, pH > 13) for 40 min. Electrophoresis was conducted at 1.2 V/cm for 30 min at 10°C. Slides were subsequently neutralized in 0.4 M Tris buffer (pH 7.5) and stained with DAPI (1 μg/mL). Fluorescence images of 100 randomly selected nucleoids per slide were acquired at 200× magnification using a Nikon Labophot-2 microscope equipped with a Pulnix TM765 CCD camera (JAI, Japan). Quantitative analysis of DNA damage was performed using Comet v.3.0 software (Kinetic Imaging Ltd., UK).

### 2.7 Animal studies

All procedures were conducted in accordance with Directive 2010/63/EU on the protection of animals used for scientific purposes and the ARRIVE Guidelines and were approved by the 2nd Institutional Animal Ethics Committee of the Medical University of Warsaw, Poland (approval No. 79/2014, dated December 16, 2014). The NUDE Balb/c mice were provided with water *ad libitum* and housed under a 12-hour light/dark cycle. The animals were acclimatized one-week prior experiments. A total of 4 × 10^6^ A549 cells, suspended in 100 µL of culture medium, were injected subcutaneously (s.c.) into the right flank of each mouse. Three weeks post-implantation, when tumors had reached an approximate size of 2–3 mm, the animals were randomly assigned to one of three groups of 7 animals in each group: untreated (Control), exposed to X-ray radiotherapy (RAD), and exposed to X-ray radiotherapy plus magnetic fluid hyperthermia (RAD+MFH).

### 2.8 Radiotherapy

X-ray radiotherapy was administered twice, with a one-week interval between each session. Prior to the RAD course, mice bearing lung tumors were intravenously injected with 125 µg of Fe@C-PEI-IgG-GOX nanoparticles containing 2.5 U of GOX. Twelve hours after the injection, the mice were subjected to radiotherapy. Irradiation was carried out using a portable X-ray device (Smart EVO 200, Yxlon International). Dosimetry and calibration of this equipment were performed using a UNIDOS electrometer (model 10001) equipped with an ionization chamber (model M30001). Dosimetry calibration was conducted by the Central Radiological Protection Laboratory – Dosimetry Calibration Laboratory for Secondary Standards, which is accredited by the Polish Centre for Accreditation (PCA), certificate number AP 057. The irradiation procedure was conducted in a room sterilized with UV radiation for 24 hours prior to the experiment. Mice were irradiated without anesthesia, individually placed in sterilized UV-irradiation trays. The irradiation parameters were as follows: total irradiation dose: 2 Gy, average dose rate: 1.18 Gy/min, current: 4.5 mA, voltage: 200 kV, distance from the X-ray tube to the subject: 30 cm.

### 2.9 Magnetic fluid hyperthermia

Magnetic fluid hyperthermia was performed six times, with one-week intervals between each hyperthermia session. The first two hyperthermia treatments were administered 24 hours after the first and second radiotherapy sessions. The remaining four hyperthermia sessions were conducted at one-week intervals. Before each MFH session, the animals received intratumoral injection of 1000 µg of iron oxide (Fe_3_O_4_) nanoparticles (Sigma-Aldrich, St. Louis, USA) and were subsequently exposed to an alternating magnetic field (AMF) for 30 minutes (f = 110.11 kHz, B = 23 mT, H = 18.3 kA·m^-1^). Further details on the iron oxide nanoparticles used, including their physicochemical properties and MFH-related characteristics, were recently published (38).

### 2.10 Magnetic resonance imaging

Preclinical *in vivo* MRI was performed on a 7 T small-animal MRI scanner (model BioSpec 70/30 USR, Bruker; Paravision 5.1) equipped with a transmit/receive birdcage resonator (model T13161V3, 40 mm inner diameter, Bruker). Animals were anesthetized with isoflurane (induction 4%, maintenance 1.5–2%), positioned supine on an MRI-compatible bed and monitored for respiration and temperature throughout scanning using small animal monitoring system (model 1025, SA Instruments). T2-weighted morphological images were acquired using the TurboRARE sequence, and angiographic images were obtained using the time-of-flight (TOF) angio-MRI technique. For T2-weighted TurboRARE imaging in the axial plane, parameters were set as follows: TR = 4300 ms, TE = 30 ms, RARE factor = 4, NA = 4, TA = 18 min 20 s 800 ms, FA = 180 deg, FOV = 30 × 30 mm, spatial resolution = 117 × 117 µm^2^, slice thickness = 0.5 mm, interslice distance = 0.6 mm, and 50 axial slices. In the coronal plane, TurboRARE imaging was performed with TR = 1800 ms, TE = 30 ms, RARE factor = 4, NA = 5, TA = 9 min 36 s, FA = 180°, FOV = 30 × 30 mm, spatial resolution = 137 × 137 µm^2^, slice thickness = 0.6 mm, slice gap = 0.1 mm, and 40 coronal slices. A 2D multi-slice gradient-echo TOF sequence (FLASH-TOF) was acquired with TR/TE = 17/4.5 ms, flip angle 70°, 4 averages, spatial resolution = 0.117 x 0.117 mm^2^, slice thickness = 0.5 mm, interslice distance = 0.3 mm, number of slices = 60, axial orientation, sequential slice excitation order. Total acquisition time was 13 min 19.68 s, and no external triggering/gating was used. The slice packages were positioned in the tumour region, in most cases in the lower abdomen. FLASH-TOF images were acquired with 40% slice overlap to improve slice-to-slice vessel continuity (39).

### 2.11 Angiography data analysis

Manual tumor segmentation was performed in 3D Slicer (v5.8.1; The Slicer Community) using TurboRARE morphological images. The resulting binary tumor masks (NIfTI format) were registered to the FLASH-TOF space for visualization purposes only. Data conversion from Paravision to NIfTI format was performed using the Bru2Nii tool (Chris Rorden/neurolabusc, GitHub). All subsequent processing and visualization steps were conducted in MATLAB (R2023; The MathWorks Inc.). The analysis workflow combined rule-based image processing with limited parameter tuning and human-in-the-loop quality assessment (QA). The main steps were as follows:

1. Whole-body masking: A body mask was generated from the FLASH-TOF magnitude images using intensity thresholding followed by basic morphological cleanup.
2. Preprocessing: Intensities within the body mask were normalized and denoised using 3D Gaussian smoothing. To improve slice-to-slice consistency, additional per-slice intensity normalization was applied.
3. Vessel-like signal enhancement: Tubular structures were enhanced using a vesselness filter (40). Complementary probability-like maps, including orthogonal-plane support maps computed as local slice-wise averages, were generated to improve spatial continuity. These maps were fused into a single probability volume using empirically selected weights. Parameter refinement was performed during method development with heuristic QA guidance but was fixed prior to group-level interpretation.
4. Segmentation and skeletonization: Vessel-like structures were obtained by thresholding the fused probability map within the body mask using a hybrid criterion combining an Otsu-derived threshold (41) with a percentile-based constraint. Small, connected components were removed, and a 3D skeleton was computed to support structural visualization.
5. Branch-level labeling for visualization: For qualitative interpretation and QA, vessel-like structures were assigned heuristic branch levels. A central trunk was identified on the skeleton, and successive branches were propagated outward using level-by-level reconstruction under exclusion constraints to prevent voxel reuse. Color coding was applied solely for visualization and descriptive purposes.

### 2.12 Statistics

All experiments were performed in triplicate unless stated otherwise, and data are presented as mean ± standard deviation (SD). The normality of data distribution was assessed using the Shapiro–Wilk test prior to inferential analysis. Statistical significance was defined as p < 0.05.

For *in vitro* cell-viability experiments (Alamar Blue assay), dose–response curves were generated and IC_50_ values calculated using nonlinear regression analysis. Group comparisons at selected concentrations and time points were performed using one-way analysis of variance (ANOVA) when normality assumptions were met, followed by appropriate *post hoc* multiple-comparison testing. When data did not meet normality criteria, non-parametric Kruskal–Wallis tests with multiple-comparison correction were applied.

For the colony-forming efficiency (clonogenic) assay, differences between experimental groups were analysed using one-way ANOVA with *post hoc* multiple-comparison testing, following verification of distribution normality. This assay was used to assess long-term proliferative capacity rather than acute cytotoxicity.

For the alkaline comet assay, quantitative DNA-damage parameters were compared between groups using one-way ANOVA or Kruskal–Wallis tests, depending on the distribution of the data, as determined by the Shapiro–Wilk test.

For *in vivo* tumour volume measurements, longitudinal tumour-growth data were analysed using two-way ANOVA, with treatment group and time as independent factors. Tukey’s *post hoc* test was applied to identify statistically significant differences between groups at individual time points. Baseline tumour volumes were confirmed to be comparable between groups prior to treatment initiation.

For survival-type data Kaplan–Meier survival curves were compared using the log-rank (Mantel–Cox) test. For multiple pairwise comparisons, p-values were adjusted using the Holm–Šídák method.

For TOF-MRI-derived endpoints, per-animal values were exported as CSV files and summarized at the group level using descriptive statistics (mean and median). Given the qualitative and visualization-oriented nature of the vascular analysis, inferential statistics were limited and interpreted cautiously. Where applicable, exploratory group comparisons were performed using parametric or non-parametric tests based on data distribution, and tumor volume was considered as a covariate for region-of-interest-based endpoints.

All statistical analyses were conducted using Statistica software (version 13.3). Tumor volume growth curves and Kaplan–Meier analyses were generated using GraphPad Prism (version 9.3.0; GraphPad Software, USA).

## 3 Results

Carbon-encapsulated iron nanoparticles, with their magnetic iron core and protective multilayer graphitic carbon shells, offer a versatile platform for biomedical applications. Over the last two decades, our group has contributed substantially to understanding their synthesis and physicochemical characterizations (29, 30, 42). Transmission electron microscopy (TEM) reveals that the pristine nanoparticles (Fe@C) without decorated surface tend to form agglomerates, while dynamic light scattering (DLS) shows that their hydrodynamic sizes in aqueous media are significantly larger due to aggregation (30). Structural analysis using X-ray diffraction (XRD) confirms the presence of body-centered cubic (bcc) α-Fe, face-centered cubic (fcc) Fe–C phases, and occasionally Fe_3_C. Raman spectroscopy further highlights the quality of the carbon shell, with raw and purified nanoparticles displaying a high G/D band intensity ratio (~1.9), suggesting a relatively ordered graphitic structure (43, 44). Functionalization, such as carboxylation, lowers this ratio (~1.5–1.6), reflecting increased surface disorder - often desirable for enhanced chemical reactivity. Surface chemistry plays a crucial role in defining the reactivity and potential applications of Fe@C nanoparticles. Infrared spectroscopy (FT-IR) and Boehm titration show that acid-treated carbon-encapsulated iron nanoparticles (e.g., Fe@C–COOH) possess significantly more acidic surface groups than unmodified ones, including carboxylic, lactonic, and phenolic functionalities (43). These modifications not only improve dispersibility but also enhance interactions with biological and environmental systems. Recent studies by Grudzinski and colleagues have focused on enhancing their stability, biocompatibility, and targeting capabilities through surface functionalization - most notably with polyethylenimine (PEI) and specific antibodies (31, 32). Functionalization with PEI results in the formation of a positively charged polymeric coating, leading to substantial modifications of the nanoparticles’ physicochemical properties, including surface charge, colloidal stability, and interactions with biological systems (33). Note that the cationic groups of PEIs are essential for covalent binding of IgG and electrostatic attachments of GOX, and therefore are integral to the structural architecture of the Fe@C-PEI-IgG-GOX nanoconstruct. Thermogravimetric analysis reveals that PEI can account for 6–52% of the total nanoparticle mass, depending on the polymer’s molecular weight and linker design (31, 32). Electron microscopy confirms the formation of a non-crystalline, amorphous PEI coating over the carbon shell. Hydrodynamic measurements show particle sizes ranging from 250 to 575 nm in aqueous suspensions, with longer linker molecules yielding more compact and stable dispersions. Zeta potential values shift to positive (ca. +30 mV), indicating enhanced colloidal stability (31, 32). Importantly, Fe@C nanoparticles with extended linker chains remain stable in suspension for over two months, highlighting the role of linker length in nanoparticle longevity. Despite these improvements, PEI-functionalized particles are known to form large protein coronas and micron-scale aggregates when exposed to biological fluids (34). Further advancement in our laboratories comes from the conjugation of monoclonal antibodies, such as anti-CD61, which confer molecular specificity to carbon-encapsulated iron nanoparticles (32, 36, 45). These antibody-decorated nanosized constructs have demonstrated selective binding to integrin-expressing melanoma and glioma cells, enabling their visualization *in vivo* using magnetic resonance imaging (32, 36, 46). Present studies on enzymatic cargos revealed the kinetic parameters of the Fe@C-PEI-IgG-GOX nanoparticles. The *K*_m_ constant, a kinetic parameter of the enzymatic reaction, was estimated at 870 µM, which is higher than that of the pristine GOX enzyme (140.5 µM). Please note that the *V*_max_ value for the tested construct was found to be 1.2 min^-1^ which is comparable with that estimated for the pristine GOX (1.46 min^-1^) (33). Although immobilization of GOX increases its Km (~870 µM vs. 140.5 µM for the free enzyme), intratumoral glucose concentrations (1–5 mM) remain well above this value, ensuring effective enzyme activity. This allows sufficient hydrogen peroxide (H_2_O_2_) generation to enhance radiosensitization, while providing a controlled tumor-starvation effect. These results confirm the enzymatic activity of the immobilized GOX on carbon-encapsulated iron nanoparticles decorated with PEI and monoclonal antibodies against CD61 integrins.

**Figure 2:**
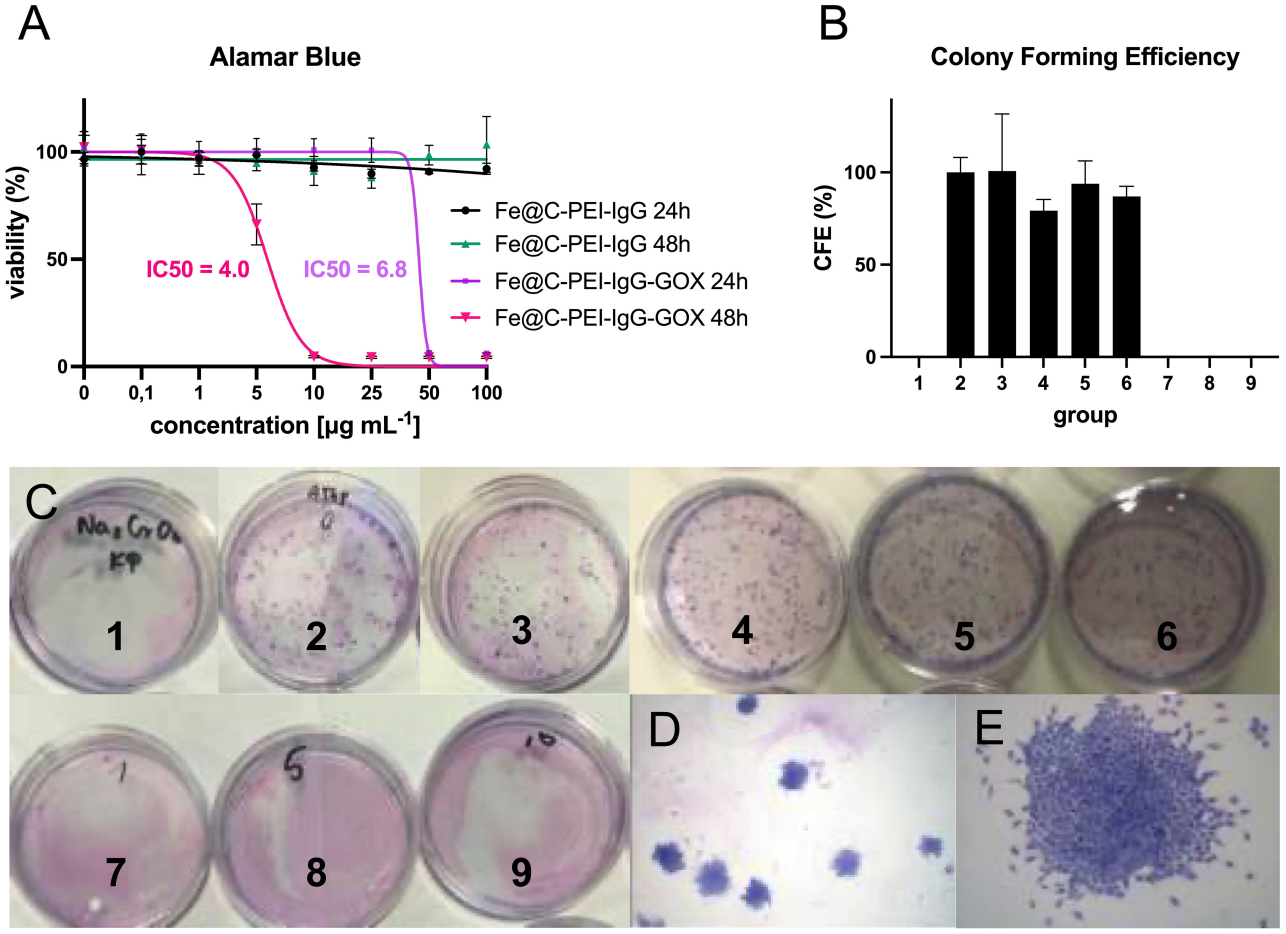
Cytotoxic effects of Fe@C-PEI-IgG and Fe@C-PEI-IgG-GOX nanoparticles on A549 cells. Cytotoxicity was evaluated using the Alamar blue assay **(A)** and colony forming efficiency (CFE) assay **(B–E)**. The treatment groups for the CFE assay were as follows: 1 – positive control (sodium chromate, Na_2_CrO_4_, 100 μM); 2 – negative control (medium); 3 – Solvent control (medium containing 10%FBS); 4 – Fe@C-PEI-IgG, 1 μg mL^-1^; 5 – Fe@C-PEI-IgG, 5 μg mL^-1^; 6 – Fe@C-PEI-IgG, 10 μg mL^-1^; 7 – Fe@C-PEI-IgG-GOX, 1 μg mL^-1^; 8 – Fe@C-PEI-IgG-GOX, 5 μg mL^-1^; 9 – Fe@C-PEI-IgG-GOX, 10 μg mL^-1^. Representative CFE assay results and A549 colonies are shown in **(C–E)**.

Evaluation of the cytotoxicity of pristine Fe@C-PEI-IgG nanoparticles without attached enzyme prepared in aqueous suspensions using the Alamar Blue assay demonstrated no cytotoxic effect on A549 cells at all concentrations used, both after 24 h and 48 h of incubation (**Figure 2A**). However, the use of enzyme-loaded nanoparticles (Fe@C-PEI-IgG-GOX) reduced the viability of A549 cells after 24 hours of incubation at concentrations of 50 μg·mL^− 1^ and 100 μg·mL^− 1^ by 93% and 100%, respectively, compared to the control group (**Figure 2A**). After 48 h of incubation with Fe@C-PEI-IgG-GOX nanoparticles, the viability of A549 cells decreased significantly at a concentration of 5 μg·mL^− 1^ (70%) and almost completely at subsequent increasing concentrations (**Figure 2A**). Based on the results from the CFE assay, a petite cytotoxic effect of pristine Fe@C-PEI-IgG nanoparticles was only observed on A549 cells, resulting in a 17% decrease in colony formation at a concentration of 1, 5 and 10 μg·mL^− 1^ with no statistically relevant differences (**Figure 2B**). In contrast, A549 cell colony formation was completely inhibited at concentrations ranging from 1 to 10 μg·mL^− 1^ of Fe@C-PEI-IgG-GOX nanoparticles (**Figures 2B–E**).

**Figure 3:**
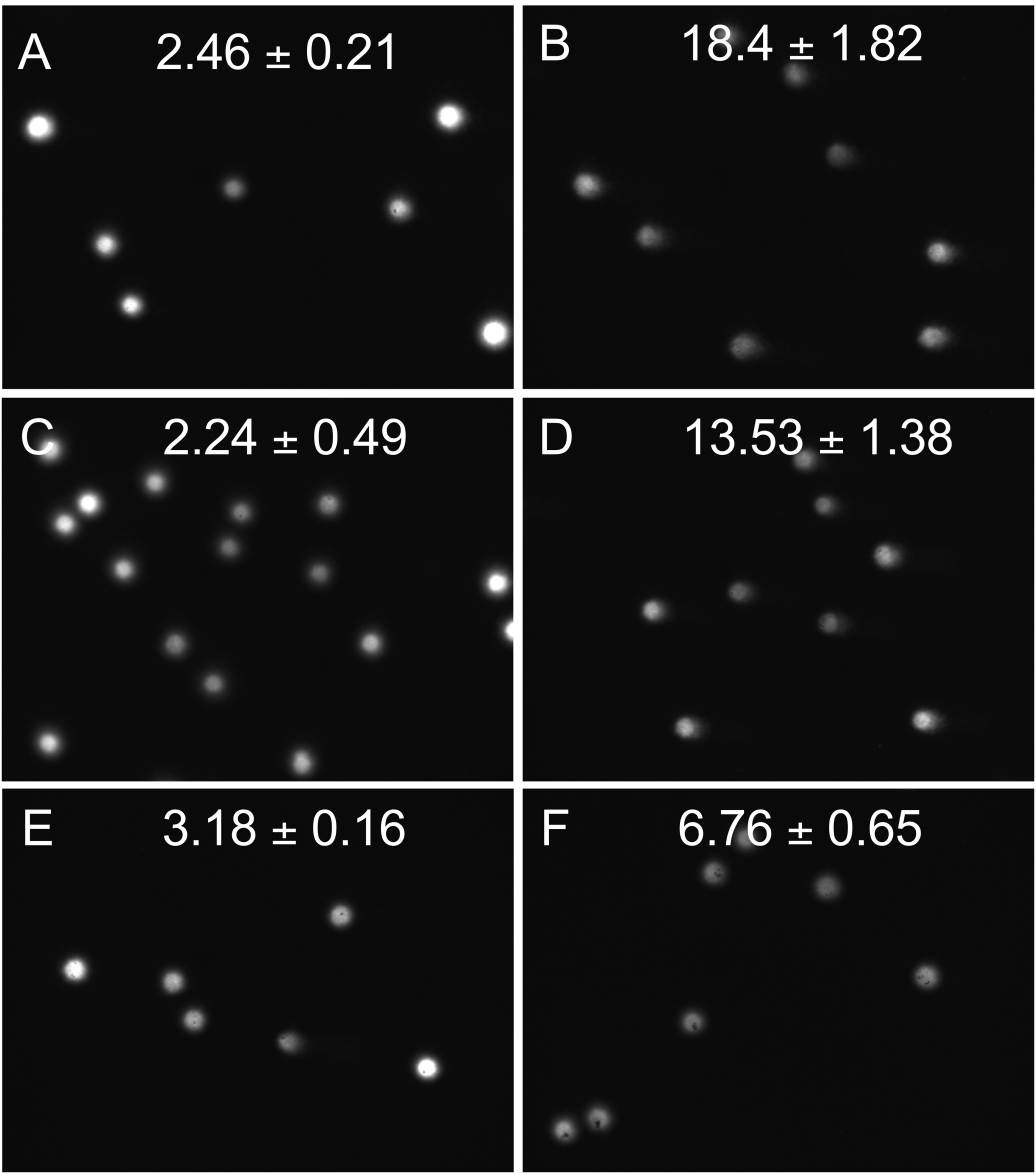
Tail Intensity (Mean ± SD) in A549 cells measured by the comet assay under different treatment conditions. **(A)** untreated control (medium); **(B)** medium + 2 Gy X-irradiation; **(C)** PBS control; **(D)** PBS + 2 Gy X-irradiation; **(E)** Fe@C-PEI-IgG nanoconjugate; **(F)** Fe@C-PEI-IgG-GOX nanoconjugate. Data represent the mean ± standard deviation (SD) of tail intensity values for each treatment condition.

The comet assay was performed to evaluate the genotoxic effects of the tested Fe@C-based nanoconjugates on A549 cells, and the resulting Tail Intensity values for each treatment condition are presented in **Figure 3**. Tail Intensity values obtained for the negative controls (medium and PBS) remained low ranging from 2.24 ± 0.21 to 2.46 ± 0.49, confirming minimal baseline DNA damage in untreated cells. Exposure of cells to 2 Gy X-irradiation, used as a positive genotoxic control, resulted in a marked increase in DNA fragmentation, with Tail Intensity values of 18.4 ± 1,82 and 13.53 ± 1.38 for medium- and PBS-treated cells, respectively, validating the sensitivity and dynamic range of the assay. Cells treated with Fe@C-PEI-IgG nanoconjugates without GOX demonstrated only a slight elevation in DNA damage compared with the control group (3.18 ± 0.16 vs. 2.46 ± 0.21), indicating that the IgG-functionalized-Fe@C-PEI platform exhibited negligible genotoxicity. A modest increase in Tail Intensity was observed following exposure of cells to Fe@C-PEI-IgG-GOX nanoparticles (6.76 ± 0.65), consistent with the expected mild oxidative stress induced by GOX-mediated H_2_O_2_ generation. Importantly, both nanoconjugate formulations produced substantially lower DNA damage than the X-ray irradiation, confirming that neither system exerts strong genotoxic effects under the tested conditions. Overall, the results demonstrate that Fe@C-based nanoconjugates, including the enzymatically active GOX-modified variant, do not significantly compromise genomic integrity in A549 cells, supporting their suitability for further biomedical applications.

**Figure 4:**
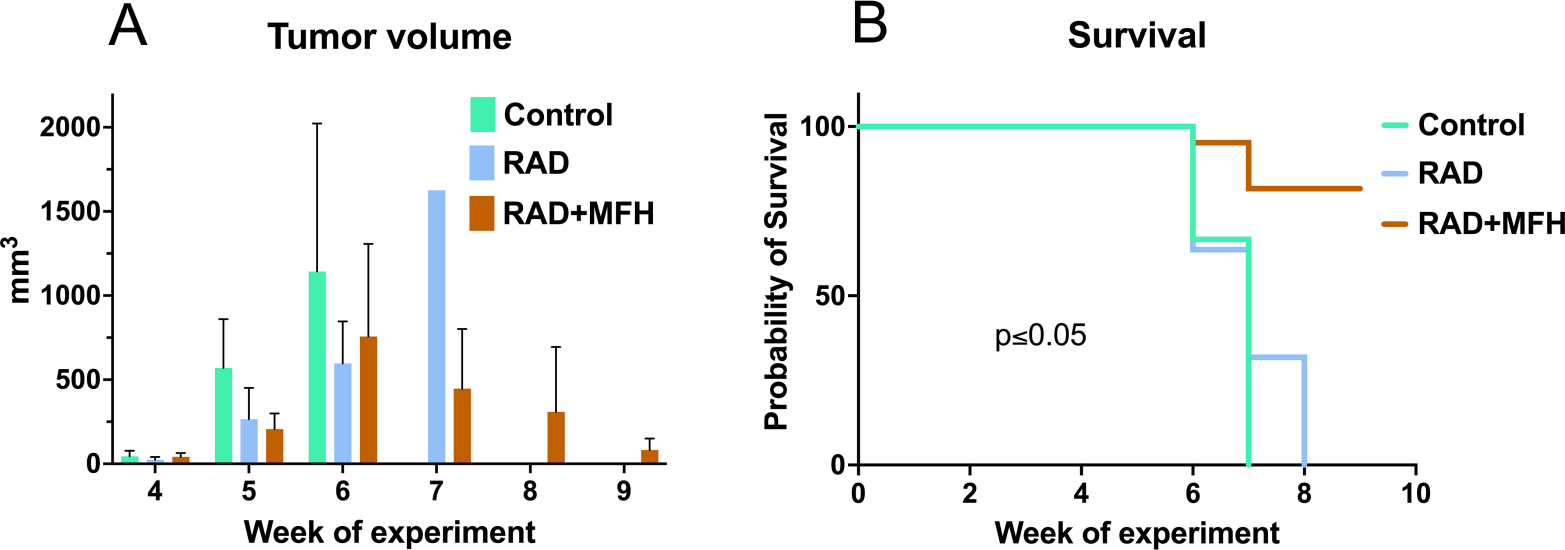
Therapeutic efficacy of radiotherapy combined with magnetic fluid hyperthermia in lung cancer (A549) xenograft NUDE Balb/c mice. **(A)** Tumor volume assessed on the selected treatment day and presented together with standard deviations, calculated for the initial group size of n = 7 animals per group: **(B)** Kaplan-Meier analysis of time to the protocol−defined humane endpoint. The endpoint was defined as a tumor size reaching 12 mm in at least one dimension, in accordance with ethical approval. Radiotherapy - RAD (total irradiation dose: 2 Gy, average dose rate: 1.18 Gy/min, current: 4.5 mA, voltage: 200 kV, distance from the X-ray tube to the subject: 30 cm), Magnetic fluid hyperthermia - MFH (f = 110.11 kHz, B = 23 mT, H = 18.3 kA·m^-1^). Non-treated NUDE Balb/c mice bearing A549 lung cancers served as controls (please see Materials and Methods for more details).

In this study, the xenograft A549 lung cancer model was used. Three weeks after the implantation of A549 cancer cells into naïve animals, the NUDE Balb/c mice bearing tumors were exposed to radiotherapy or a combination of radiotherapy and magnetic fluid hyperthermia. Untreated tumor-bearing mice were served as controls. All animals, treated and untreated, were monitored weekly for general health and tumor size (**Figure 4A**). Iron oxide nanoparticles were injected directly into the tumor prior to hyperthermia sessions to ensure sufficient material for effective heating. The first radiotherapy and magnetic fluid hyperthermia treatments were applied three weeks post-implantation (week 4 of the experiment), when tumor sizes were comparable across all groups, with no statistically significant differences observed (44.8 ± 33.1 mm^3^ in the control group, 43.6 ± 17.7 mm^3^ in the RAD-treated group, and 41.3 ± 23.2 mm^3^ in the RAD+MFH-treated group). In control mice that did not receive any treatment, tumor volumes reached the critical threshold size of 12 mm in at least one dimension, which was used as the exclusion criterion, much earlier than in the treated groups (**Figure 4B**). According to animal welfare regulations, mice with tumors exceeding this size were removed from the study. As a result, all control mice were excluded by week 7. In the RAD-treated group, all animals were excluded by week 8. In contrast, in the RAD+MFH-treated group, only three animals were excluded throughout the entire experiment, showing the lowest mortality among all groups. The experiment was terminated at week 9. Multiple comparisons of Kaplan–Meier survival curves revealed no significant difference between the control group and the RAD-only group, whereas both the RAD+MFH group versus control and the RAD+MFH group versus RAD-only group showed statistically significant differences in survival (p = 0.002).

**Figure 5:**
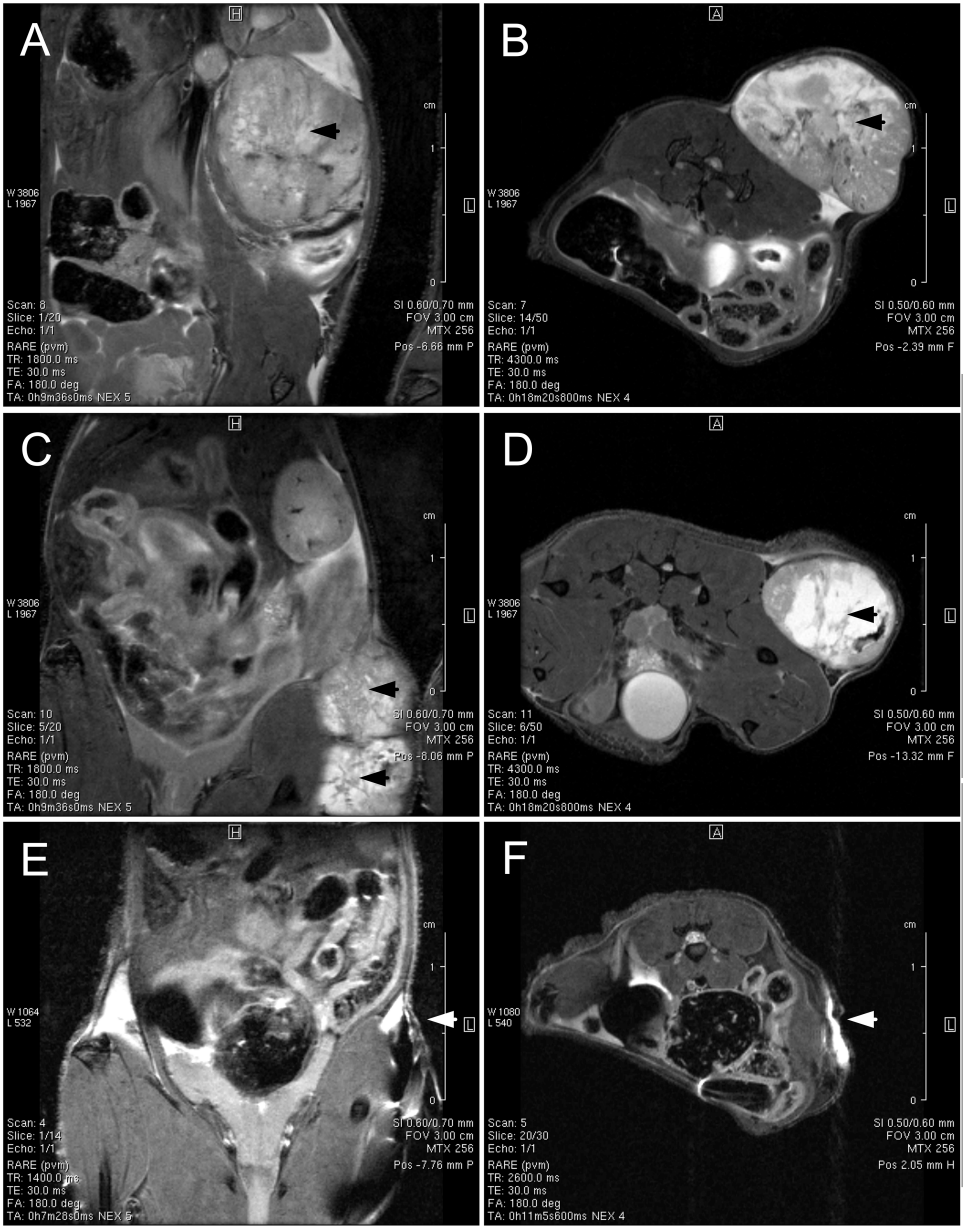
Representative T2 images of NUDE Balb/c mice bearing human lung cancer (A549) from the control **(A, B)**, radiotherapy **(C, D)**, and radiotherapy plus magnetic fluid hyperthermia **(E, F)** groups. The tumor appears as a large mass located on the flank of the untreated mouse **(A, B)**. Following radiotherapy, two distinct tumors are observed in the mouse **(C, D)**. A small tumor mass is visible in the animal exposed to radiotherapy combined with magnetic fluid hyperthermia **(E, F)**. The tumor location is indicated by arrows. Anatomic imaging was conducted using a Bruker BioSpec 70/30 USR 7 T system running ParaVision 5.1 using T2-weighted TurboRARE sequences. In the coronal plane **(A, C, E)**, the acquisition parameters were set as follows: TR = 1800 ms, TE = 30 ms, RARE factor = 4, NA = 5, TA = 9 min 36 s, FA = 180°, FOV = 30 × 30 mm, spatial resolution = 137 × 137 µm^2^, slice thickness = 0.6 mm, slice gap = 0.1 mm, and 40 coronal slices. In the axial plane **(B, D, F)**, the acquisition parameters were set as follows: TR = 4300 ms, TE = 30 ms, RARE factor = 4, NA = 4, TA = 18 min 20 s 800 ms, FA = 180 deg, FOV = 30 × 30 mm, spatial resolution = 117 × 117 µm^2^, slice thickness = 0.5 mm, interslice distance = 0.6 mm, and 50 axial slices.

**Figure 6:**
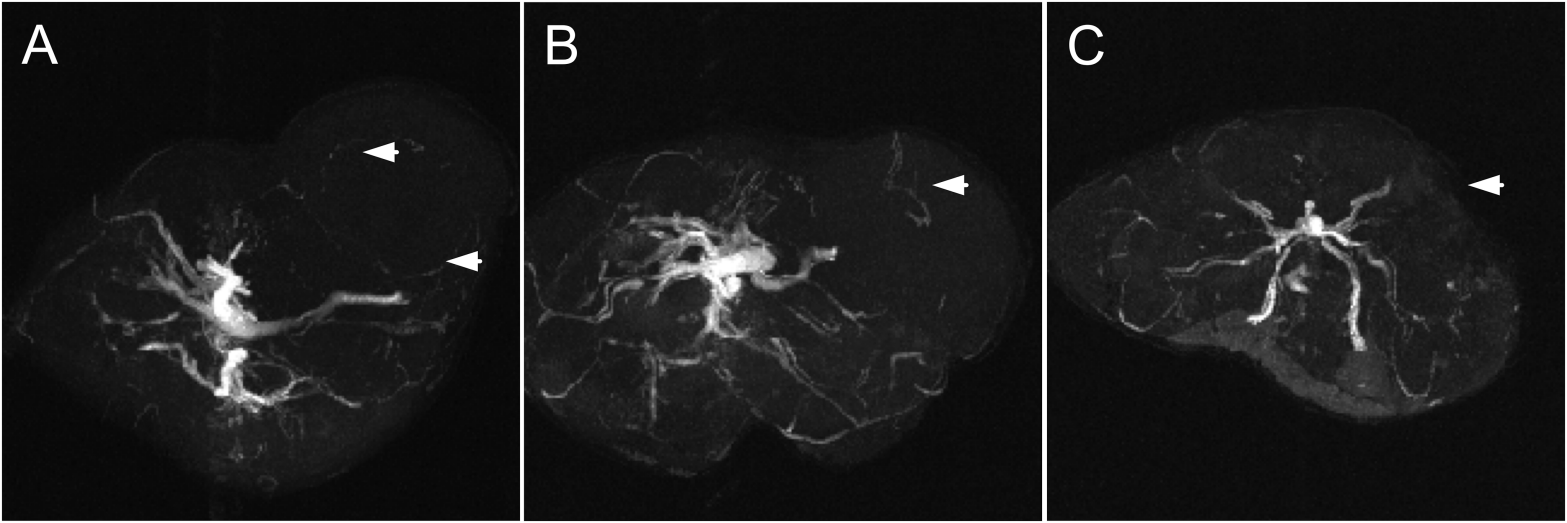
Representative angiography images of NUDE Balb/c mice bearing human lung cancer (A549) from the control **(A)**, radiotherapy **(B)**, and radiotherapy plus magnetic fluid hyperthermia **(C)** groups. The tumor location is indicated by arrows. Angiography imaging was conducted using FLASH time-of-flight (TOF) MRI on a Bruker BioSpec 70/30 USR 7 T system running ParaVision 5.1 using the Bruker FL2D ANGIO (FLASH-TOF) 2D multi-slice gradient-echo sequence. Data were acquired in axial orientation with TR/TE = 17/4.5 ms and flip angle = 70°, using 4 averages. Coverage comprised 60 slices with 0.5 mm slice thickness and 0.3 mm slice distance (i.e., 0.2 mm overlap). The in-plane field of view was 30×30 mm², reconstructed to 256×256, giving an in-plane resolution of 0.117×0.117 mm²; the encoding matrix was 256×192 in phase-encode.

Studies evidenced that mice subjected to radiotherapy and magnetic fluid hyperthermia treatment showed a significant tumor-growth suppression compared to the control non-treated mice (**Figure 3A**). Moreover, in this group we observe tumor growth until the 6th week, while from the 6th week onwards, the tumor sizes decrease, which does not occur in the group subjected to radiotherapy only. The Kaplan-Meier survival curve (**Figure 4B**) highlights the differences in survival between the control group and the groups receiving RAD alone or RAD followed by MFH. Analysis of the results revealed significant variations in survival times between the treatment groups and the control group. The survival time for the control group was the shortest, lasting only 7 weeks, whereas the RAD group had a survival time that was one week longer. The highest survival rate was observed in the group treated with both RAD and MFH. These results indicate that animals in the RAD+MFH group had significantly longer survival times (p < 0.05). Furthermore, the survival curve for this group remained higher for a longer duration, suggesting that the combination of treatments may be associated with a better prognosis. These findings were also evidenced on magnetic resonance imaging showing the tumor and its vasculature on T2-weighted and MRI-TOF images (**Figures 5**, **6**). The T2-weighted images in the coronal and axial planes provided valuable insights into the anatomical structures and tumor characteristics in NUDE Balb/c mice bearing human lung cancer. In the coronal plane (**Figure 5A**), the control animal exhibited a well-defined tumor with significant tissue swelling and distortion of surrounding structures, typical of the untreated cancer progression. In contrast, the radiotherapy-treated mouse (**Figure 5C**) showed a slightly reduction in tumor volume and less surrounding tissue distortion, suggesting that radiotherapy had a noticeable effect on tumor growth. The animal exposed to RAD and MFH (**Figure 5E**) demonstrated even greater tumor shrinkage and fewer signs of local invasion, indicating a potentially synergistic effect of combined treatment modalities. Similarly, the axial images (**Figures 5B, D, E**) supported these findings, with tumors in the control group presenting as larger and more irregular in shape, compared to the more compact and reduced tumor size observed in both the RAD and RAD+MFH animals. These results imply that radiotherapy, particularly in combination with magnetic fluid hyperthermia, may significantly impact tumor morphology, potentially delaying or inhibiting cancer progression.

**Figure 7:**
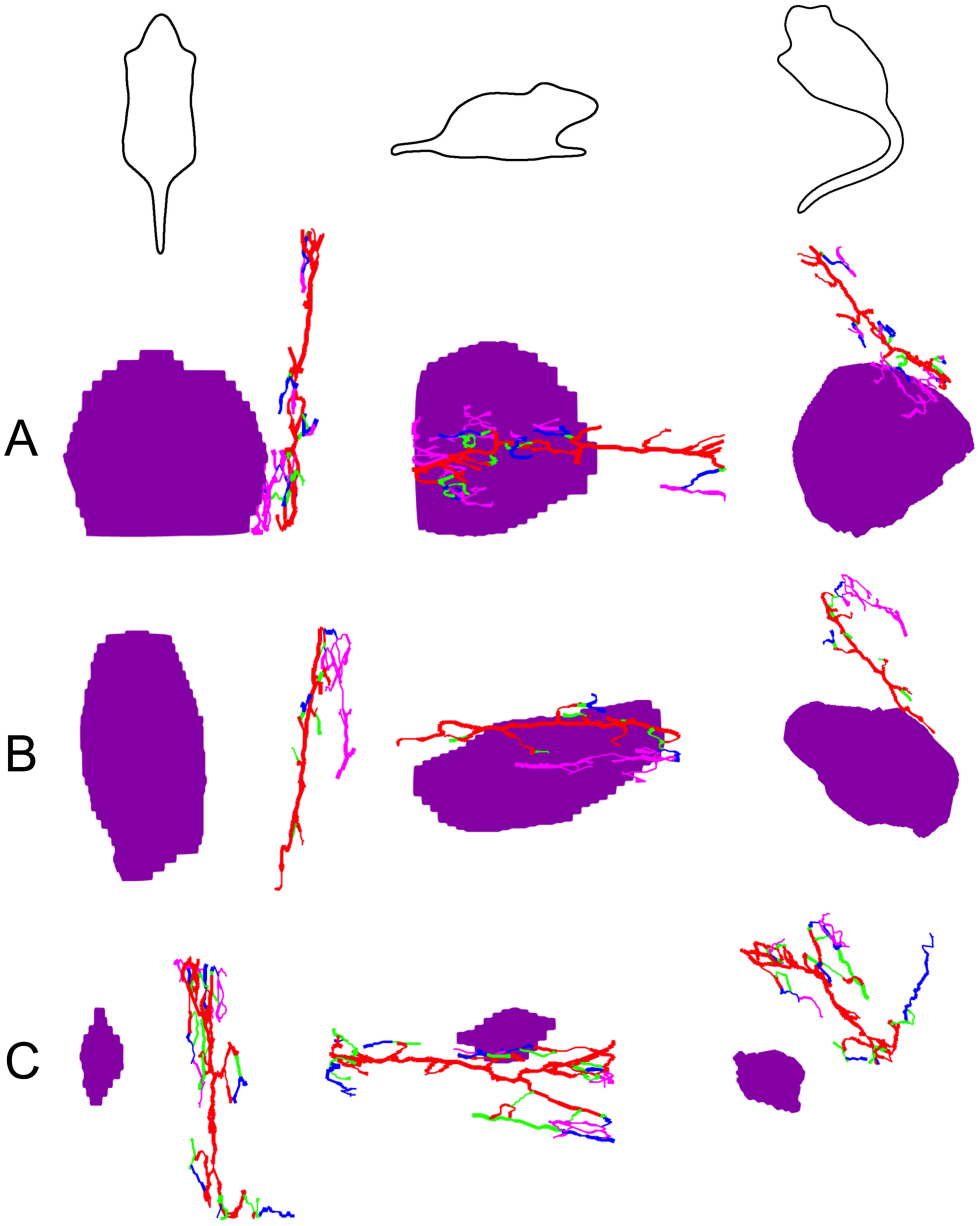
Qualitative visualization of branch-level vessel labeling in FLASH-TOF angiography. A 3 × 3 panel presents representative renderings of whole-body TOF-MRI vessel segmentation and branch-order visualization across three experimental groups. Rows correspond to control **(A)**, radiotherapy **(B)**, and radiotherapy plus magnetic fluid hyperthermia **(C)**. Columns show three standardized viewpoints as showed by mouse outline icons: top view (left), side view (middle), and angled/perspective view (right). The segmented vascular network is color-coded by hierarchical branching level: the main trunk (primary vessel path) is colored red; first-order branches emerging directly from the trunk is colored green; second-order branches emerging from green branches is colored blue; and higher-order branches is colored magenta. The tumor mask is colored purple. These panels are generated by an in-development qualitative analysis pipeline for iterative method refinement and visual QA.

Magnetic resonance angiography (angio-MRI) was used to support qualitative assessment of tumor-associated vascular morphology. In control animals, the tumor vasculature appeared highly irregular, with numerous vessel-like structures, consistent with aggressive angiogenic patterns (**Figure 6A**). In the radiotherapy group, angio-MRI suggested partial attenuation of the vascular network, in line with known radiation-associated effects on tumor blood supply (**Figure 6B**). In contrast, animals treated with radiotherapy followed by magnetic fluid hyperthermia exhibited a more pronounced reduction in visibly perfused vessel-like structures within the tumor region (**Figure 6C**). These observations were qualitatively consistent with vessel-like structure reconstructions derived from three-dimensional volumetric datasets (**Figures 7A–C**). Segmentation-based visualizations were generated to assist interpretation of vascular architecture and were explored using an interactive, human-in-the-loop quality-assessment application. The software enabled visualization of color-coded vessel-like structures overlaid on derived maps (orthogonal slices, MIP views, fused probability maps, and 3D renderings) and supported heuristic scoring during method development. These qualitative assessments were used solely to guide parameter selection prior to group-level interpretation. Three-dimensional reconstructions demonstrated visually identifiable central vascular trunks with accompanying peripheral branches, with apparent differences in branch density and complexity between groups. In animals subjected to radiotherapy followed by magnetic fluid hyperthermia, fewer peripheral vessel-like structures and reduced apparent topological complexity were observed compared with the control and radiotherapy-only groups (**Figures 7A–C**). These imaging features qualitatively corresponded with reduced flow-related signal intensity on angio-MRI within the tumor region. Importantly, vessel extraction and branch-level labeling in the present study were intended for qualitative visualization and descriptive assessment rather than objective quantification. Therefore, while the qualitative imaging findings are consistent with alterations in tumor-associated vascular patterns following treatment, definitive conclusions regarding vascular remodeling or angiogenic activity would require dedicated quantitative imaging endpoints and statistical validation.

One has to be noted that the current vessel extraction and branch-level labeling should be interpreted as a qualitative assessment. While major trunks are often recovered, smaller vessels may be missed, and some voxels may be assigned to an incorrect branch level. This is consistent with known challenges in 3D angiography segmentation, including extreme class imbalance and the limited availability of accurately annotated 3D reference data (47). The level labels are heuristic and are currently intended for qualitative QA rather than as an anatomical taxonomy. Since FLASH-TOF uses sequential slice excitation with slice overlap, some crosstalk/saturation may occur, potentially reducing inflow enhancement and sensitivity to small vessels. We addressed this issue by tracking slice-to-slice intensity and applying per-slice intensity normalization within the body mask.

## 4 Discussion

The main challenge in radio-oncology is achieving effective tumor destruction while minimizing harm to healthy tissues. Although radiotherapy plays a crucial role in treating lung cancer, its success can be hindered by the radioresistance of cancer cells and the risk of damaging nearby healthy lung tissues. Therefore, the use of magnetic nanoparticles (MNPs), offer promising avenues to enhance the efficacy of RAD for lung cancer. MNPs, especially those with high Z (atomic number) elements, can enhance the absorption of X-rays, leading to a localized increase in the dose delivered to the cancer cells (48–50). This intensified energy deposition can generate more free radicals (e.g., reactive oxygen species) (51) and directly induce more complex and irreparable DNA double-strand breaks, which are critical for cell death (52). Beyond inducing more damage, some MNPs have been shown to interfere with the cellular DNA repair pathways. By hindering the cancer cells’ ability to repair radiation-induced damage, MNPs effectively amplify the cytotoxic effects of RAD (52). In our study the comet assay results demonstrate that the Fe@C-based nanoconjugates exhibit a favorable genotoxicity profile in A549 cells. Both negative controls yielded low Tail Intensity values, confirming minimal intrinsic DNA damage in untreated cultures and validating the experimental conditions. As expected, X-irradiation at 2 Gy also induced a pronounced increase in DNA strand breaks, highlighting the sensitivity of the assay and providing a robust reference for evaluating the genotoxic potential of the nanoconjugates. Importantly, the Fe@C-PEI-IgG nanoconjugates without GOX induced only a marginal increase in DNA damage compared with the negative controls, suggesting that surface functionalization with PEI and IgG only does not compromise genomic integrity under the tested conditions. Although PEI is known to exert cytotoxic and genotoxic effects at high molecular weights or concentrations, its immobilization on a carbon-encapsulated iron core appears to mitigate these risks by restricting direct polymer–DNA interactions. This observation supports the concept that nanoplatform engineering can effectively reduce undesired PEI-mediated toxicity while preserving functional performance. Interestingly, the Fe@C-PEI-IgG-GOX nan(bio)material produced slightly higher Tail Intensity values than the Fe@C-PEI-IgG variant, which is consistent with the enzymatic activity of GOX. One has to be emphasized that glucose oxidase catalyzes the oxidation of glucose with concomitant generation of hydrogen peroxide (H_2_O_2_), which is a well-known mediator of oxidative DNA lesions and widely used as a positive control in the comet assay. Nevertheless, the observed degree of DNA damage remained low and substantially below that induced by X-ray radiation (2 Gy), indicating that GOX-based oxidative stress was mild and did not result in overt genotoxic damages. These results align with reports showing that moderate levels of ROS generated by enzyme-functionalized nanomaterials can modulate cellular processes without causing extensive genomic injuries. Taken together, the findings suggest that both nanoconjugate systems (Fe@C-PEI-IgG and Fe@C-PEI-IgG-GOX) are generally safe with respect to DNA integrity, with the GOX-modified construct showing only a modest increase in genotoxic stress attributable to its catalytic function. The low level of DNA damage observed for both formulations supports their further development for biomedical applications, particularly those involving targeted delivery, catalytic therapies, or redox-based tumor modulation. Future studies will be necessary to explore long-term genomic stability, systemic dose- and time-dependent effects, and *in vivo* responses, which will provide a more comprehensive understanding of the safety profile of these Fe@C-based nanoplatforms. The incorporation of the alkaline comet assay provides direct experimental evidence that enhanced DNA damage contributes to the therapeutic efficacy of the combined treatment. The significant increase in DNA strand breaks observed following X-ray radiotherapy combined with magnetic fluid hyperthermia and the multifunctional nanoplatform is consistent with impaired DNA damage repair and increased vulnerability of tumor cells to genotoxic stress. Importantly, DNA damage represents a converging endpoint for several interacting mechanisms present in this system. Iron-based nanoparticles are well established as radiosensitizers that amplify radiation-induced oxidative stress through catalytic reactive oxygen species (ROS) generation, resulting in increased DNA lesions and reduced clonogenic survival (53). Concurrently, magnetic fluid hyperthermia has been shown to disrupt DNA damage response pathways and impair repair kinetics, thereby potentiating radiation-induced genotoxicity, as described in classical and clinical studies by Horsman and Overgaard (54) and by Dewhirst and colleagues (55). In parallel, glucose oxidase-mediated glucose depletion is expected to weaken cellular antioxidant defenses and energy-dependent DNA repair pathways, thereby exacerbating oxidative DNA damage. Numerous studies have demonstrated that GOX-based tumor starvation strategies promote hydrogen-peroxide accumulation, redox imbalance, and heightened sensitivity to additional cytotoxic stressors, including radiotherapy and hyperthermia (26, 27). Taken together, the alkaline comet assay results indicate that X−ray irradiation and GOX−loaded nanoparticles may contribute to the accumulation of persistent DNA damage. Moreover, given that magnetic fluid hyperthermia has been reported in the literature to induce DNA alterations, its inclusion in the combined treatment may influence the overall DNA damage response, although this effect was not directly assessed in the present study. While the inclusion of additional molecular markers, such as γ-H2AX or apoptosis-related proteins, would allow a more detailed pathway-level analysis, the present findings suggest that DNA damage accumulation may contribute to the observed therapeutic effects and warrants further mechanistic investigation.

Magnetic nanoparticles are known to modulate cell cycle progression, often leading to phase-specific arrest (e.g., G2/M), which enhances the susceptibility of cells to radiation (52, 53). They can also directly or indirectly trigger apoptotic pathways, leading to programmed cell death (52). In some cases, MNPs might improve oxygenation within hypoxic (low oxygen) tumor regions. Hypoxia is a significant factor in radioresistance, as oxygen is crucial for the formation of DNA-damaging free radicals during RT. By ameliorating hypoxia, MNPs can indirectly enhance radiosensitivity (48, 56). Please note that nanoparticles, after iv administration can accumulate within tumor cells through enhanced permeability and retention (EPR) effect or by targeted delivery mechanisms (48). Functionalization of nanoparticles with targeted ligands, such as monoclonal antibodies, significantly improves their ability to selectively bind to specific molecular and cellular targets in cancer cells (57). In our study, we used magnetic nanoparticles functionalized with a monoclonal IgG antibody to target integrin αvβ3, which is often abundant on the surface of lung cancer cells as well as on newly formed blood vessels (angiogenesis) supplying tumors (24, 58). The choice of a monoclonal IgG antibody as the target ligand was based on its ability to specifically block the β3 subunit (CD61) of the αvβ3 integrin receptor, which is overexpressed in lung cancer as well as in several other tumors, e.g., glioma (58, 59). Integrin αvβ3 plays a key role in tumor cell adhesion, migration, and angiogenesis, making it an attractive target for cancer diagnosis and therapy (60). By promoting the survival and migration of endothelial cells, it helps tumors acquire the necessary blood supply (61). Moreover, integrins, including αvβ3, mediate the adhesion of cancer cells to the extracellular matrix (58). This interaction is crucial for cancer cells to detach from the primary tumor, invade surrounding tissues, enter the bloodstream, and eventually form secondary tumors (metastasis) in distant organs (61). By functionalizing MNPs with monoclonal IgG antibody, we aimed to selectively bind these nanoparticles to αvβ3 integrin-expressing tumor cells, thereby improving the specificity of nanoparticle targeting to αvβ3 integrin-expressing tumor cells, which helps reduce off-target effects and increase the efficacy of radiotherapy. Once internalized, these nanoparticles can act as radiosensitizers, meaning they increase the sensitivity of cancer cells to radiation. In our previous studies, we observed a marked increase in the concentration of carbon-encapsulated iron nanoparticles functionalized with a monoclonal antibody (anti-CD61) against the β3 subunit in rat glioma tissues, which may be related to the EPR effect and the targeted delivery mechanism following intravenous administration of these nanoparticles (36). A limitation of the present study is the use of a single non-small cell lung cancer (NSCLC) cell line (A549) for both *in vitro* and *in vivo* experiments. Although this widely used model enables consistent evaluation of treatment effects, it does not capture the biological heterogeneity of NSCLC, which may limit the generalizability of the findings to other lung cancer subtypes or molecular backgrounds.

While MRI confirmed tumor-associated accumulation of the magnetic nanoplatform in mice bearing A549 tumors, quantitative biodistribution analysis was not performed in the present study and therefore represents an acknowledged limitation. It should be emphasized that non-invasive imaging techniques such as MRI provide valuable spatial information on nanoparticle localization, but do not allow reliable discrimination between active receptor-mediated targeting and passive accumulation driven by the enhanced permeability and retention effect (62–64). Accumulating evidence indicates that, following systemic administration, the dominant mechanism governing tumor accumulation of nanomedicines at the whole-organ level is EPR-mediated extravasation, whereas surface-conjugated targeting ligands typically exert a more pronounced influence on cellular internalization, intratumoral retention, and biological activity rather than on absolute tumor uptake (65, 66). This phenomenon is further influenced by rapid formation of a protein corona in the bloodstream, which can partially mask targeting ligands and modulate nanoparticle–cell interactions *in vivo* (67). In this context, anti-CD61 functionalization of the presented nanoplatform should be interpreted primarily as a strategy to enhance interactions with αvβ3-expressing tumor cells and tumor-associated vasculature following extravasation, rather than as a determinant of exclusive tumor-specific organ-level accumulation. Such ligand-mediated interactions are expected to improve intracellular delivery and local retention of GOX, thereby contributing to the observed therapeutic efficacy even in the absence of biodistribution data. From a broader perspective, although negatively charged nanoparticles are frequently associated with prolonged circulation times and improved tumor penetration at the tissue level, it is now well recognized that whole-tumor accumulation of nanomedicines following systemic administration is predominantly governed by EPR effect and is largely independent of surface charge (65, 66). In this framework, surface charge becomes particularly relevant after extravasation, where interactions with tumor cells and subsequent intracellular internalization are critical determinants of biological activity. Accordingly, in the present nanoplatform, the positive zeta potential arising from PEI functionalization was intentionally introduced to facilitate efficient biomolecule conjugation and to enhance cellular uptake. Positively charged nanocarriers are known to exhibit stronger electrostatic interactions with negatively charged cellular membranes, promoting endocytosis and intracellular delivery (68, 69). This property is of particular importance for glucose oxidase-based starvation therapy, as the enzymatic activity of GOX requires close intracellular or pericellular proximity to glucose pools in order to induce effective metabolic disruption and oxidative stress. Consequently, the positive surface charge of the developed nanoparticles should be interpreted not as an attempt to maximize passive tumor accumulation, but as a functional design element aimed at improving post-accumulation cellular delivery and therapeutic performance, while maintaining sufficient colloidal stability under physiological conditions. In addition to the magnetic nanoparticles (Fe@C-PEI-IgG-GOX), the glucose oxidase enzyme was intravenously dosed prior to radiotherapy, thereby introducing a metabolic stress component complementary to radiotherapy and magnetic fluid hyperthermia. However, one thing should be taken into account when administering nanoparticles in this way. When a nanomaterial is administered intravenously, it inevitably encounters a complex biological environment composed of numerous plasma proteins and other blood components. As is well established in the field of nanobiotechnology, virtually all nanoscale materials undergo spontaneous adsorption of plasma proteins upon exposure to blood, forming what is referred to as the protein corona. This process is dynamic, competitive, and highly context−dependent, influenced by particle size, surface charge, morphology, and the biochemical composition of plasma. Consequently, it is not possible to assume *a priori* whether the Fe@C-PEI-IgG formulation (or its GOX-modified variant) would bind to specific blood components, nor can we precisely predict the molecular weight of any resulting complexes. In principle, several features of our material could contribute to interactions with plasma proteins. The PEI layer contributes a positive surface charge, which is known to facilitate electrostatic association with negatively charged proteins such as albumin or fibrinogen. The presence of IgG on the particle surface may partially shield the underlying nanostructure, potentially decreasing nonspecific interactions, but IgG itself may participate in competitive exchange with other plasma proteins. The carbon coating may also promote adsorption of hydrophobic proteins. In the case of the GOX−functionalized form, the attached enzyme introduces an additional protein layer that can further modulate the overall interaction profile. Nevertheless, the composition and extent of protein adsorption depend on numerous physicochemical and biological variables that are impossible to fully predict without targeted experimentation. Importantly, even if a corona does form, its mass and composition cannot be described by a single molecular weight value. Different proteins vary widely in size, from albumin (~66 kDa) to immunoglobulins (~150 kDa) to fibrinogen (~340 kDa), and the number of proteins adsorbed per nanoparticle fluctuates depending on environmental conditions, local concentrations, and temporal evolution of the soft and hard corona layers. Protein corona formation is neither static nor stoichiometric: proteins exchange over time, may partially unfold upon binding, and adsorb in different orientations. Thus, any complex formed between Fe@C-PEI-IgG (or its GOX form) and plasma proteins would not possess a unique or definable molecular weight. Such a value can only be determined experimentally under specific biological conditions, for instance using techniques such as dynamic light scattering, SDS-PAGE analysis, or proteomics−based corona characterization. For these reasons, the mentioned interactions are real and scientifically relevant, yet inherently variable and context−specific. We therefore respectfully maintain that, at this study, the possibility of nanoparticles binding to blood proteins cannot be ruled out, but further research is required to clarify this issue.

In the present study, cytotoxicity assays showed lower survival rates of lung cancer (A549) cells treated with Fe@C-PEI-IgG-GOX nanoparticles, suggesting increased treatment efficacy due to the presence of GOX, which produces hydrogen peroxide. In cancer cells, which have a high glucose requirement, GOX activity leads to local glucose depletion (the so-called starvation therapy) and the generation of reactive oxygen species (ROS), especially H_2_O_2_, which damage cancer cells (70). These ROS can cause additional damage to tumor cells, potentially enhancing the efficacy of radiotherapy (56, 71). Overproduction of H_2_O_2_ causes massive oxidative stress, leading to loss of mitochondrial potential, activation of caspases (-9 and -3), and initiation of the intrinsic apoptosis pathway (72, 73). This ultimately triggers cancer cell death (72, 74). Other drug-conjugated nanomaterials (e.g., gold, carbon) exhibit indirectly similar effects-increased ROS, decreased mitochondrial potential, caspase activation, and a stronger cytotoxic effect than the free drug (74, 75). However, it is worth emphasizing that GOX nanocarriers overcome the intrinsic limitations of the free enzyme, such as immunogenicity and short circulation half−life, and enable its stable and localized delivery to target cells, thereby increasing selectivity toward cancer cells (26).

The administration of iron oxide nanoparticles before an alternating magnetic field (AMF) is well established and represents the fundamental mechanism behind the therapeutic effect. When exposed to an AMF, iron oxide nanoparticles dissipate heat through Néel and Brownian relaxation processes, enabling localized thermal ablation of tumor tissue without damaging surrounding healthy regions. Thus, the presence of iron nanoparticles inside the tumor is essential for generating the controlled hyperthermic effect. In the clinical and preclinical magnetic hyperthermia literature, iron oxide nanoparticles are typically administered directly into the tumor tissue, often via intratumoral injection (19, 38). This mode of delivery maximizes local nanoparticle concentration and minimizes systemic distribution. Because the particles remain physically confined within the tumor mass, there is very low probability of significant systemic elimination before the application of AMF or before any subsequent administration of other therapeutic nanosystems such as Fe@C-PEI-IgG or Fe@C-PEI-IgG-GOX. Our studies show that intratumorally injected iron oxide nanoparticles tend to remain localized for prolonged periods, particularly when embedded within dense tumor stroma, where their mobility is limited (76). Regarding toxicity, iron oxide nanoparticles are often described as relatively biocompatible, although their potential toxic effects have been widely investigated and discussed in the literature (77, 78). They have been used in clinical applications (e.g., as MRI contrast agents), and their toxicity is generally considered to be dose-dependent and relatively acceptable under controlled conditions. Furthermore, their degradation products can enter physiological iron metabolism pathways, which may contribute to their favorable safety profile. This is one of the reasons why iron oxide systems are often considered among the preferred materials for magnetic fluid hyperthermia. It should be noted that engineered carbon-encapsulated iron nanoparticles (Fe@C-PEI-IgG and Fe@C-PEI-IgG-GOX) behave differently from simple iron oxide nanoparticles. Their elimination plausibly depends on several factors including surface chemistry, protein corona formation, hydrodynamic diameter, and degree of aggregation. In general, nanoparticles in this size range are removed through the mononuclear phagocyte system (MPS), particularly by macrophages in the liver and spleen. Subsequent processing may involve lysosomal degradation, partial dismantling of organic components (e.g., PEI or protein layers), and eventual slow clearance of inorganic residues. If any fraction of the material reaches the bloodstream, hepatic and splenic uptake would represent the predominant route of long-term elimination. Adverse reactions, if they occur, are typically associated with transient inflammatory responses or oxidative stress, but such effects are usually mild and have been reported only at significantly higher doses than those used in targeted or intratumoral strategies. For the Fe@C-based nanosystems developed in our work, we expect adverse reactions to be minimal due to the protective carbon shell, the stabilizing PEI layer, and the selective targeting provided by IgG. Nevertheless, comprehensive quantitative biodistribution studies, including organ-level iron quantification and tumor-to-organ ratios using e.g. ICP-MS, will be required in future investigations to fully validate targeting specificity and to support translational development of this nanoplatform. As with any nanomaterial intended for biomedical use in humans, the full toxicological profile must be also evaluated experimentally.

Magnetic hyperthermia is widely recognized to enhance radiotherapy primarily through inhibition of DNA double-strand break repair, modulation of tumor oxygenation, and induction of heat-associated cellular stress responses, rather than through direct cytotoxicity alone. Similarly, iron-based nanoparticles are established radiosensitizers that amplify radiation-induced reactive oxygen species generation and promote ferroptotic cell death pathways. In parallel, GOX-based tumor starvation strategies have repeatedly demonstrated synergistic enhancement of both radiotherapy and hyperthermia via glucose depletion and oxidative stress (53, 79, 80). Accordingly, the present *in vivo* study was experimentally designed to evaluate the integrated therapeutic efficacy of the multifunctional nanoplatform under clinically relevant combination treatment conditions, while minimizing animal use in compliance with ethical guidelines and the principles of reduction. One has to be noted that the combination of radiotherapy with magnetic fluid hyperthermia introduces an additional layer of therapeutic synergy (81). MFH relies on the application of an alternating magnetic field to magnetic nanoparticles internalized within tumor tissue, resulting in localized heat generation and a controlled temperature increase (typically 40–45 °C). This localized hyperthermia acts cooperatively with ionizing radiation to sensitize tumor cells and enhance therapeutic outcomes. Before the MFH courses, animals were given intratumorally iron oxide nanoparticles (15 nm). Direct injection into the tumor, ensuring high local concentration of these nanosized agents, which allow generate heat after external AMF application. The generated heat raises the temperature of the tumor tissue to hyperthermic ranges (82, 83). This controlled hyperthermia can further augment the radiosensitizing effects of MNPs. The temporal relationship between radiotherapy and hyperthermia is a critical determinant of the underlying mechanisms of therapeutic cooperation. Classical radiobiological studies have demonstrated that hyperthermia induces the strongest radiosensitizing effect when applied concurrently with, or within approximately one hour of, irradiation. Under these conditions, hyperthermia directly inhibits DNA double-strand break (DSB) repair pathways, particularly homologous recombination, resulting in increased fixation of radiation-induced DNA damage (55, 84). In the present study, magnetic fluid hyperthermia was applied 24 hours after irradiation, a timing that does not correspond to the canonical window for maximal DSB repair inhibition. This delayed application is therefore unlikely to reflect classical, DNA repair-driven radiosensitization alone. However, hyperthermia is increasingly recognized as a multimodal adjuvant therapy whose biological effects extend beyond acute interference with DNA repair kinetics. These include sustained modulation of tumor blood flow and oxygenation, induction of heat-shock proteins, enhancement of oxidative stress, and increased vulnerability of metabolically stressed tumor cells (54). Notably, radiation itself induces long-lasting alterations in tumor physiology, including inflammatory signaling, vascular remodeling, and metabolic perturbations. Hyperthermia applied at later time points may therefore interact with this altered post-irradiation tumor microenvironment, resulting in cooperative or additive therapeutic effects even in the absence of direct DSB repair inhibition. From this perspective, the observed enhancement in the combined radiotherapy and MFH group likely represents a composite effect arising from delayed radiosensitization, microenvironmental modulation, and stress-mediated tumor cell vulnerability. The 24-hour interval was intentionally selected to approximate a sequential and clinically feasible treatment paradigm, rather than an experimentally optimized radiosensitization window. While this approach does not isolate the contribution of individual radiobiological mechanisms, it provides insight into the therapeutic potential of MFH as a practical adjuvant to radiotherapy under conditions that more closely resemble clinical scheduling constraints (85). Future studies incorporating multiple hyperthermia time points relative to irradiation, as well as direct assessment of DNA damage markers and microenvironmental changes, will be necessary to systematically delineate timing-dependent mechanisms of synergy. Please note that the magnetic fluid hyperthermia itself is cytotoxic to lung cancer cells (19, 38). Elevated temperatures can directly denature proteins, damage cellular membranes, and induce apoptosis, making the tumor cells more vulnerable even before radiation is applied (19, 38). Moreover, treatment with MFH led to noticeable changes in metallomic profiles and glycan structures in lung cancer cells. These effects were accompanied by the release of matrix metalloproteinases (MMP-1, MMP-2, and MMP-9) and an increase in cell membrane permeability, suggesting that MFH primarily disrupts membrane integrity and alters interactions between the cell and the extracellular matrix (76). Hyperthermia can induce apoptosis (programmed cell death) or necrosis in tumor cells (86) and what more important, MFH can additionally enhance the effectiveness of radiotherapy and chemotherapy in different ways. Heat is a potent inhibitor of DNA repair enzymes. When combined with radiotherapy, MFH can further compromise the cancer cells’ ability to repair radiation-induced DNA damage. Additionally, the heat can increase the generation of free radicals from water within the cells, further contributing to DNA damage (22, 87). Mild hyperthermia can transiently increase blood flow to the tumor (22), potentially improving oxygen delivery and mitigating hypoxia, thereby enhancing the oxygen enhancement ratio of RT (56, 88, 89). Emerging research suggests that MFH can induce an immunogenic cell death, leading to the release of danger-associated molecular patterns (DAMPs) and tumor-associated antigens (90, 91). This can prime an anti-tumor immune response, potentially converting an immunologically “cold” tumor into a “hot” one, thereby complementing the effects of RT and MNPs (92, 93). Furthermore by damaging tumor cells and their microenvironment, hyperthermia can inhibit tumor progression (86). In overall, the results obtained in this study clearly confirmed the higher effectiveness of lung cancer treatment when X-ray irradiation and magnetic fluid hyperthermia were combined, especially when using monoclonal antibody (anti-CD61)-functionalized carbon-encapsulated iron nanoparticles additionally loaded with glucose oxidase. Although additional control groups would be required to more precisely delineate the individual contributions of magnetic fluid hyperthermia, iron-related radiosensitization, and GOX-associated metabolic effects, these processes have been reported previously in independent studies and serve as a contextual framework for interpreting the observed treatment effects. In the future, it is anticipated that the integration of magnetic fluid hyperthermia with radiotherapy and immunotherapy will open new therapeutic avenues for the personal treatment of highly aggressive and molecularly resistant lung cancers.

## 5 Conclusions

This study explores a novel multimodal therapeutic strategy combining X−ray irradiation with magnetic fluid hyperthermia, based on bioengineered carbon−encapsulated iron nanoparticles functionalized with polyethyleneimine, anti−CD61 monoclonal antibodies, and glucose oxidase (Fe@C−PEI−IgG−GOX). The efficacy of this approach was evaluated in A549 lung cancer models both *in vitro*, using A549 cell cultures, and *in vivo*, using NUDE Balb/c mice bearing A549 xenograft tumors. The Fe@C−PEI−IgG−GOX nanoparticles markedly reduced A549 cell viability and completely suppressed colony formation *in vitro*, accompanied by increased DNA damage as assessed by the alkaline comet assay. When combined with X−ray radiotherapy and magnetic fluid hyperthermia *in vivo*, this multimodal treatment resulted in enhanced therapeutic efficacy and a prolonged time to the protocol−defined endpoint compared with control conditions. Collectively, these findings indicate improved treatment performance of the proposed combined regimen in the A549 lung cancer xenograft model. The biological effects observed are consistent with mechanisms previously reported for similar multifunctional nanoplatforms, including metabolic stress associated with glucose oxidase activity, oxidative stress−mediated radiosensitization, ligand−mediated interactions with lung cancer cells, and heat−induced cytotoxicity generated during magnetic fluid hyperthermia. However, intratumoral glucose depletion, reactive oxygen species or hydrogen peroxide generation, as well as detailed thermal dosimetry, were not directly quantified in the present study. Accordingly, these processes should be considered putative contributing factors rather than experimentally validated mechanisms within this model. The experimental design was intentionally focused on evaluating the overall therapeutic efficacy of a combined multimodal treatment strategy integrating bioengineered carbon−encapsulated iron nanoparticles, magnetic fluid hyperthermia, and radiotherapy. This approach was informed by prior *in vitro* findings demonstrating that the Fe@C−PEI−IgG nanoplatform lacking glucose oxidase exhibited negligible cytotoxicity and did not impair clonogenic survival, supporting the prioritization of GOX−containing nanoparticles for *in vivo* evaluation. Nevertheless, the absence of additional control groups (*in vivo*) limits the ability to disentangle the relative contributions of hyperthermia, iron−mediated radiosensitization, and enzymatic metabolic perturbation. Consequently, the present findings should be interpreted primarily in terms of integrated treatment response rather than definitive mechanistic validation. Despite these limitations, the results support the potential of this combined therapeutic approach as a promising platform for future studies incorporating more detailed mechanistic analyses.

## Data Availability

The raw data supporting the conclusions of this article will be made available by the authors, without undue reservation.

## Glossary

MFH: Magnetic fluid hyperthermia
PEI: Polyethyleneimine
GOX: Glucose oxidase
RAD: Radiotherapy
IARC: International Agency for Research on Cancer
MNPs: Magnetic nanoparticles
ROS: Reactive oxygen species
ATCC: American Type Culture Collection
PBS: Phosphate-buffered saline
PCA: Polish Centre for Accreditation
AMF: Alternating magnetic field
MRI: Magnetic Resonance Imaging
TEM: Transmission electron microscopy
DLS: Dynamic light scattering
XRD: X-ray diffraction
EPR: Enhanced permeability and retention
DAMPs: Danger-associated molecular patterns
DSB: DNA double-strand break.
